# Attitudes of medical students toward psychiatry in Eastern Mediterranean Region: A systematic review

**DOI:** 10.3389/fpsyt.2022.1027377

**Published:** 2023-01-26

**Authors:** Mohammad Mohebbi, Nastaran Nafissi, Farzaneh Ghotbani, Arash Khojasteh Zonoozi, Hossein Mohaddes Ardabili

**Affiliations:** ^1^Student Research Committee, Faculty of Medicine, Mashhad University of Medical Sciences, Mashhad, Iran; ^2^Psychiatry and Behavioral Sciences Research Center, Faculty of Medicine, Ibn-e-Sina Hospital, Mashhad University of Medical Sciences, Mashhad, Iran

**Keywords:** psychiatry, stigma, attitudes, medical students, Eastern Mediterranean Region (EMR)

## Abstract

**Background:**

Psychiatry is facing one of the highest levels of shortages among medical specialties. Stigma toward psychiatry plays an influential role in medical students' decision to choose psychiatry as a career and has been reported to be prevalent in different parts of the world, particularly in low/middle-income countries.

**Objective:**

To systematically review the Eastern Mediterranean Region (EMR) medical students' attitudes toward psychiatry, to assess whether their attitudes are stigmatized or not, and the factors affecting their attitudes.

**Method:**

PubMed, Scopus, Web of Science, and PsychInfo (PsycARTICLES) were searched using a combination of main terms “stigma,” “psychiatry,” “medical students,” and the name of Eastern Mediterranean countries. Cross-sectional studies assessing the attitudes of EMR medical students toward psychiatry were included in this review.

**Results:**

Ten studies were eligible to be included in the result synthesis. These were from Pakistan (*n* = 3), Iran (*n* = 2), Saudi Arabia (*n* = 1), Lebanon (*n* = 1), Egypt (*n* = 1), Bahrain (*n* = 1), and Oman (*n* = 1). Most studies reported a combination of both positive and negative attitudes among medical students; however, the overall attitude was positive. Factors affecting medical students' attitude toward psychiatry included poor psychological well-being, having a friend with a psychiatric illness, involving in a romantic relationship with someone suffering from mental illness, young age, frequency of exposure to psychiatry clerkship/teaching, and quality of psychiatry clerkship. Nevertheless, the final positive or negative outcome of these factors on students' attitudes remained controversial.

**Conclusion:**

Considering the lack of sufficient data from most EMR countries, we need to exercise caution in interpreting the results of this review. Nevertheless, our review indicates that psychiatry is not stigmatized among EMR medical students, and they have generally positive attitudes toward it. The findings of studies evaluating influencing factors are contradictory and demand further exploration.

## Introduction

Mental health, according to WHO, is conceptualized as “a state of well-being in which the individual realizes his or her own abilities, can cope with the normal life stress, can work productively and fruitfully, and is able to make a contribution to his or her community” (1). There are, however, alterations from this state in one out of four individuals (2, 3). The magnitude of psychiatric disorders has been under intense scrutiny. The Global Burden of Diseases, Injuries, and Risk Factors Study 2019 (4), sheds light on the considerable burden of mental disorders, being among the top ten causes of burden globally, with no evidence of improvement since 1990. The current COVID-19 pandemic has further complicated the situation. There are reports of increased psychiatric disease incidence, along with exacerbations of preexisting mental disorders associated with pauses or changes in the patients' routine care during the pandemic (5–7). This significant and growing burden necessitates an enhanced level of alertness. However, psychiatry is experiencing a “recruitment crisis” across the world, especially in low/middle-income countries, not to mention Eastern Mediterranean Region (EMR) (8–12). This crisis has markedly exacerbated the existing *treatment gap*, without being effectively addressed by worldwide policymakers.

Psychiatry is facing one of the highest levels of shortages among medical specialties (13). Considering the UK alone, the royal college of psychiatrists is calling for 7,000 more places in medical schools (14), and the US is expected to experience a 21,000 shortage of psychiatrists by 2030 (15). The extent of the situation, however, varies greatly among nations, with as low as 0.1 psychiatrists and 1 psychiatrist per 100,000 population in low-income and EMR countries, respectively, compared to more than 8 psychiatrists per 100,000 population in high-income countries (16).

Among multiple factors influencing the decision to choose psychiatry as a career, perception of psychiatry plays an essential role (17). Stigma toward psychiatry has been reported to be prevalent in different parts of the world, particularly in low/middle-income countries (18–20). Psychiatry has been perceived to be less scientific and prestigious, with lower treatment efficacy than other specialties (17, 21). Awareness of this stigmatized view has prompted worldwide researchers to investigate the attitudes of medical students toward psychiatry in an attempt to deepen the comprehension of the situation and influencing factors. Accordingly, factors affecting medical students' choice of career and attitude toward psychiatry include but no limited to the quality of psychiatric clerkship, the perceived attractive lifestyle of psychiatrists, the improvement seen in affected individuals after treatment, the influence from role models, family and personal history of mental illness, and certain personality traits (e.g., openness to experience) (22–25). Notably, some studies have highlighted the influence of cultural, social, and regional factors (19, 26, 27).

WHO's EMR contains 22 countries with a population of 645 million and distinct socioeconomic and health challenges (28). Prolonged emergencies have disabled the health systems of some of these countries while affecting most other neighboring nations. This may be reflected in the substantially high prevalence of mental illnesses and substance use disorders in these countries (29). Barriers to universal health coverage, health workforce maldistribution and availability, and issues related to rural workforce retention all indicate incompetent policies and provoke growing alarm regarding the state of health systems in the EMR region (30). Therefore, these countries need active support to develop national plans and achieve the United Nations Sustainable Development Goals.

A review of international medical students' attitudes toward psychiatry found highly negative opinions toward psychiatry as a career (19). This finding is supported by Lyons, who observed the same pattern in global medical students (18). However, little is known about the attitudes of EMR medical students toward psychiatry and it is not clear which factors may influence their attitudes.

In order to develop requisite policies tackling mental health issues, documentary evidence of students' stigma toward psychiatry and related factors is an absolute obligation for every country. Considering EMR countries and the challenges they face within the realm of mental health disorders, providing such evidence becomes even more critical. Hence, given the abovementioned uncertainties about EMR medical students, we aim to systematically review the studies focusing on the attitude of medical students toward psychiatry in EMR and the factors affecting it.

## Methods

### Pre-registration and search strategy

The protocol of this systemic review was registered on the Open Science Framework (OSF) registry (Registration 10.17605/OSF.IO/3M2UW). The systematic review followed Preferred Reporting Items for Systematic Reviews and Meta-Analyses guidelines (PRISMA 2020) (31). We searched PubMed, Scopus, Web of Science, and PsycINFO (PsycARTICLES). In addition, reference lists of all eligible publications were searched using citation tracking sources (Google Scholar) to ensure a comprehensive search. We started searching on March 20, 2022 and updated it toward the end of the review. The last search occurred on May 6, 2022. No filter was applied regarding the language, type, or publication year of the articles in the search strategy. We used a combination of the main terms “stigma,” “psychiatry,” “medical students,” and the names of each EMR country (32) in all the above-mentioned databases. The search strategies for all databases are available in Supplementary material (Supplementary Tables 1–4).

### Eligibility criteria

Studies with the following criteria were included in this review. The same criteria were applied for both phases of the selection process (title/abstract screening and full-text review):

- Population: We included studies targeting undergraduate medical students and excluded studies involving residents, graduated medical students, medical doctors, and other groups not considered undergraduate medical students.
- Outcome: We included studies evaluating the attitudes of medical students toward psychiatry in EMR.
- Context: We included studies focusing on WHO's EMR countries (32), including Afghanistan, Bahrain, Djibouti, Egypt, Iran, Iraq, Jordan, Kuwait, Lebanon, Libya, Morocco, Oman, Pakistan, Palestine, Occupied Palestine Territory, Qatar, Saudi Arabia, Somalia, Sudan, Syrian Arab Republic (Syria), Tunisia, United Arab Emirates, and Yemen.
- Types of studies: We included cross-sectional studies and excluded other study designs such as review articles, cohorts, case controls, and clinical trials, but reference lists of review articles were checked for eligible studies. Non-English and non-Persian publications were excluded in the screening process.

### Selection process

For the purpose of de-duplication, record screening, and other citation management processes, reference management software was used.

The title and abstract of the reports were screened by two independent reviewers (FG, MM). Studies fulfilling inclusion criteria or having any uncertainty regarding their eligibility were considered for full-text review. Following title/abstract screening, two reviewers (NN, MM) independently assessed the full texts of the reports against the inclusion criteria and recorded the reasons for exclusion at this stage. Consensus or referring to a third reviewer (FG) resolved the controversies. Identifying information of the studies was visible to the screeners.

### Data collection process

Two investigators (NN, MM) extracted the required data using a standardized Excel spreadsheet, and a third verified the process (FG). We conducted a calibration exercise to maximize consistency among reviewers. Corresponding authors were contacted if there was any missing information.

Extracting variables were established through discussion. We extracted the following data: article characteristics (e.g., first author, country of origin, year of publication), response rate, mean age, number of participants, number of male participants, scales used for assessing the stigma/attitudes, mean scale score, predictors of the stigma/attitudes, limitations, and the main results.

We used mean scores of the questionnaires and standard deviation to interpret and present results. By an online tool (33), we pooled the results of those studies using the original version of the ATP-30 questionnaire and reported both mean scores and standard deviations.

### Risk of bias (quality) assessment

Joanna Briggs Institute's (JBI) critical appraisal checklists for analytical cross-sectional and prevalent studies (34, 35) were used for risk assessment in this review. After a calibration session, two independent reviewers (NN, MM) assessed the quality of the studies. Controversies were resolved by discussion or referring to a third party. The JBI Critical Appraisal Tools were used to assess the methodological quality of studies and to determine the extent to which they have addressed bias possibility. For prevalence studies, the JBI checklist includes nine questions regarding sample frame, sampling process, sample size, setting description, condition identification and measurement, statistical analysis, and response rate. Point 1 is for “yes” answers, while point 0 is for “no,” “unclear,” or “not applicable” answers. After discussion, the authors set a threshold of 5 for prevalence studies to be included in the review. For analytical cross-sectional studies, the JBI checklist includes eight items with questions regarding inclusion criteria, setting description, confounding factors, statistical analysis, exposure, condition, and outcome measurement. The authors agreed on a threshold of 4 for the inclusion of analytical cross-sectional studies.

## Results

### Study selection

**Figure 1:**
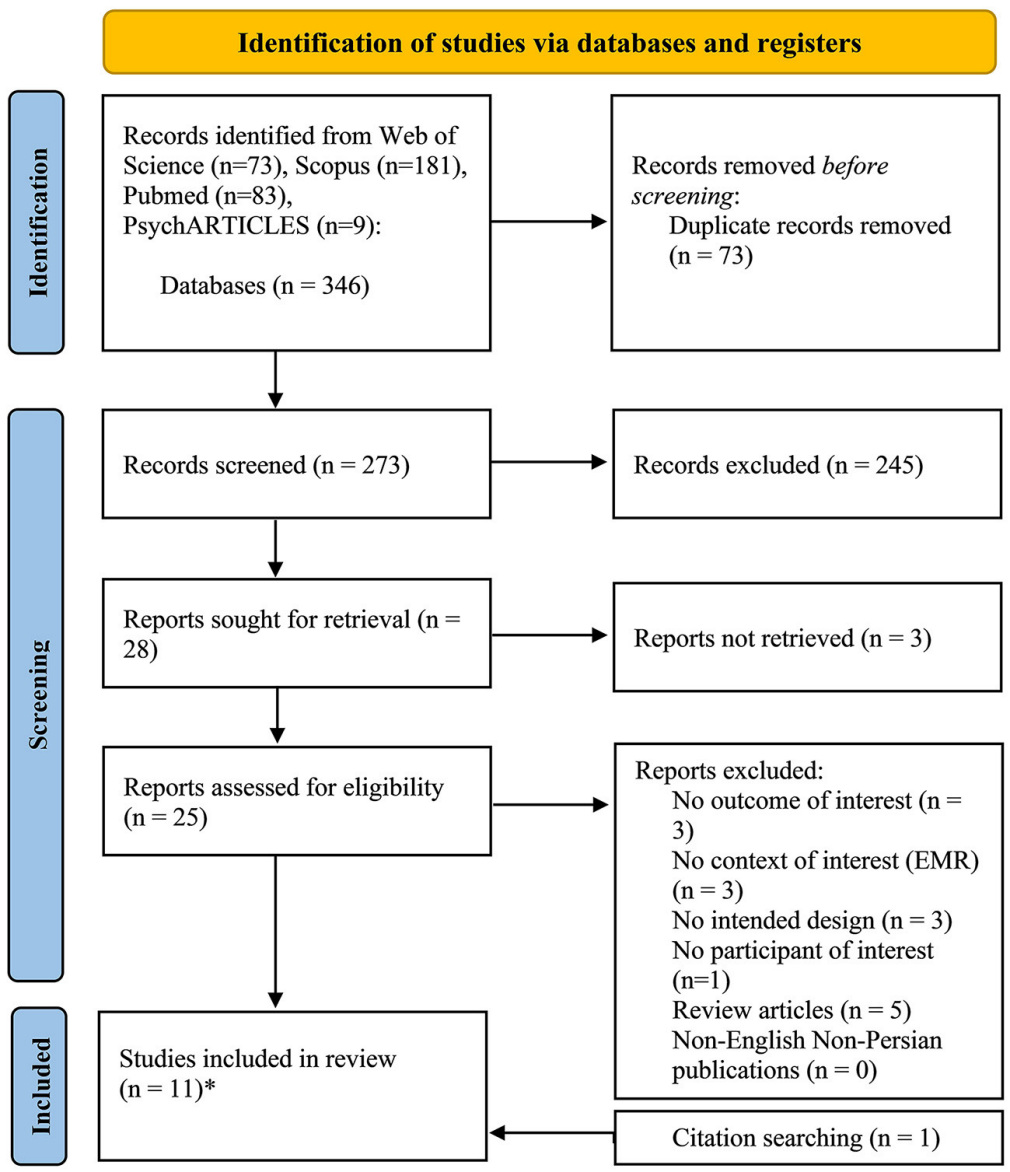
The PRISMA diagram presenting the procedure of literature searching and selection with numbers of articles at each stage. *One of these studies was not included in the final discussion due to the risk of bias regarding our review question.

The systemic search of databases resulted in 346 citations. Following deduplication (*n* = 73), 273 records underwent title/abstract screening, of which 29 studies were considered for full-text review. Finally, considering one additional report retrieved through searching reference lists of included studies and related reviews, the searching process produced 11 studies. Excluding one report due to the risk of bias, a total of 10 reports were included in the final discussion. Figure 1 depicts the PRISMA diagram of the selection process. We excluded pre/post surveys assessing the effect of psychiatry clerkship on the medical students' attitudes toward psychiatry (22, 36–42) because their primary aim was not in line with this review, and their different study designs would have contributed to increased heterogeneity of included studies.

### Characteristics of studies

For assessing the attitudes of medical students, the most frequently used measurement was the Attitudes Toward Psychiatry-30 items (ATP-30) questionnaire (*n* = 9) (43–51). Toudehskchuie et al. (49, 50) customized the questionnaire for their pre-clinical participants and omitted items related to psychiatric knowledge and teaching. Other measurements included Attitudes Toward Psychiatry-18 items (ATP-18) (52) and a questionnaire adapted from Feifel questionnaire (27). The study using the former questionnaire was not included in the final discussion due to the risk of bias regarding our review question. This study was an international survey containing one of the EMR countries (Iraq) but did not provide details about the response of Iraqi students, and hence was prone to the risk of bias with regard to our review question.

ATP-30 is a widely used questionnaire consisting of 30 items introduced by Burra et al., with adequate validity, reliability, and good internal consistency (Cronbach's alpha = 0.874) (53). It has four major sections: attitudes toward psychiatric patients and psychiatric illness, psychiatrists and psychiatry, psychiatric knowledge and teaching, and finally psychiatric treatment and hospitals. Answers to the questions are provided based on a five-point Likert scale (strong disagreement, disagreement, neutral, agreement, strong agreement). Scores above 90 indicate a positive attitudes, scores below 90 indicate negative attitudes, and 90 demonstrates a neutral attitude. One of the studies used an adapted version of the Feifel questionnaire, consisting of 24-item and a few open-ended questions (27). The items ask about the demographic background of the participants, students' perceived important factors in the choice of a specialty, students' priorities of specialty choice, and students' opinions regarding different aspects of specialties such as financial reward, lifestyle, job satisfaction, challenges, prestige in the medical community, prestige in the general public, bright future, scientific foundation, etc. The scoring was based on a five-point Likert scale from 1 = very attractive to 5 = extremely unattractive. There is no reliability/validity data available for this questionnaire (27, 54).

**Figure 2:**
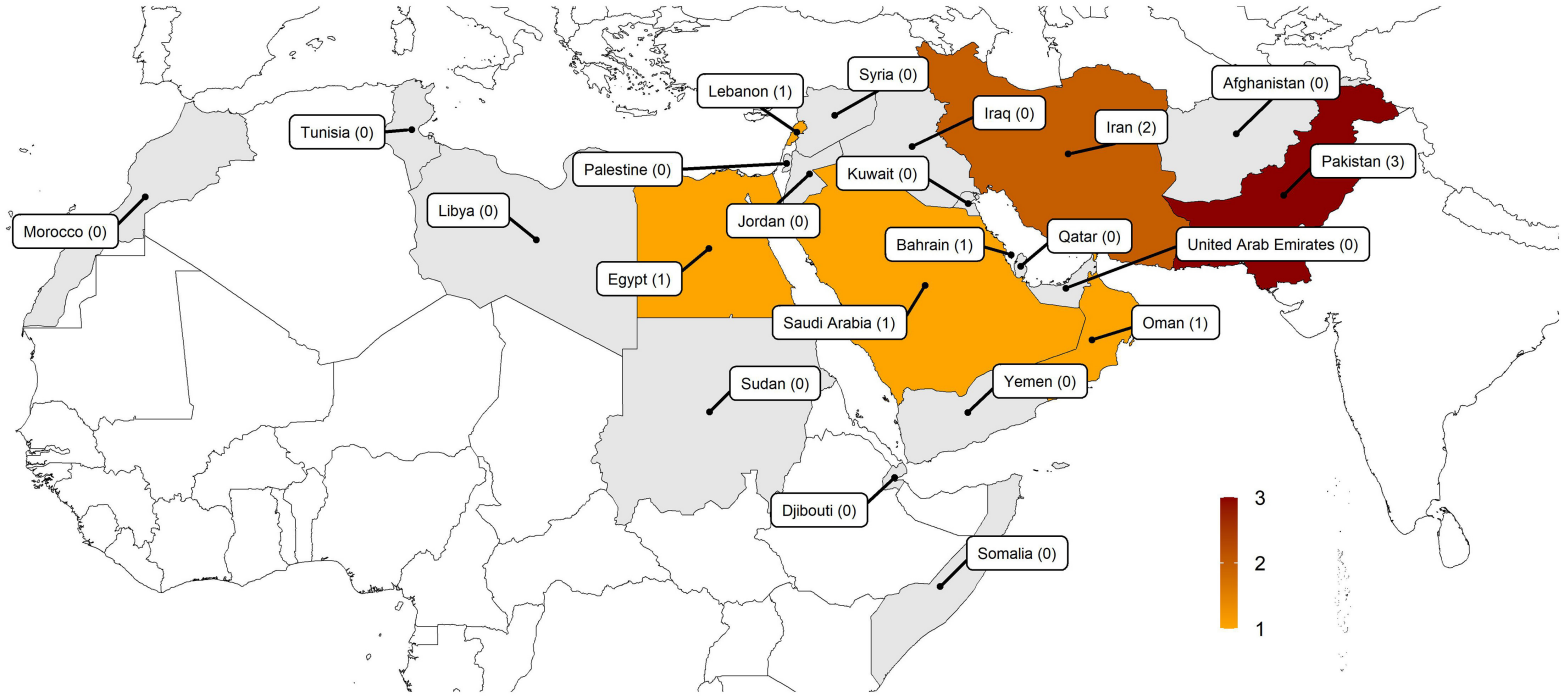
Geographical distribution of the included studies (the cross-sectional studies conducted in the Eastern Mediterranean Region reporting the attitudes of medical students toward psychiatry).

Regarding the geographical distribution of the reports, the studies were conducted in seven different countries within the EMR; three studies came from Pakistan (27, 44, 48), two from Iran (49, 50), and others from Saudi Arabia, Lebanon, Egypt, Bahrain and Oman (*n* = 5) (43, 45–47, 51). Figure 2 depicts the geographical distribution of the reviewed studies.

The sample size ranged from 130 to 635, with a total population of 3,567. Two studies included only clinical final year medical students (27, 44), one included pre-clinical students (49), and others included a combination of both clinical and pre-clinical students (43, 45–48, 50, 51). About twenty medical schools participated in the surveys. Seven studies conducted uni-center surveys (43, 46–51) and others, excluding one that did not specify (52), conducted multicenter surveys with a minimum of 2 centers and a maximum of 7 (27, 44, 45). The response rate ranged from 11% to 100%, with rates of over 80% in seven studies (63% of total studies).

Seven studies adopted the convenient sampling technique and recruited the participants in a classroom, before or after lectures, or in the hospitals, before or after rotations (27, 44, 46–51). One study adopted voluntary response sampling (45) and conducted an online survey. Another study did not specify the sampling method and how the questionnaires were distributed (43). With regard to publication year, the most recent report was published in 2021 (45), and the oldest report dates back to 2002 (47). Four studies were published in 2016 or afterward (43, 45, 46, 51), while others were published before this year. Table 1 shows the characteristics of the reviewed studies.

**Table 1:** The general characteristics of the included articles in the review of studies assessing the stigma toward psychiatry among medical students in the Eastern Mediterranean Region.

**First author**	**Country**	**Sample size**	**Response rate (%)**	**Male respondents**	**The academic year of the study population**	**Age, mean** **(Standard Deviation)**	**Scale**
El Hage et al. (45)	Lebanon	607	10.83	257 (42.7%)	1–7	NM	ATP-30
Alzahrani (43)	Saudi Arabia	317	100	121 (38%)	Mixed	22.4 (1.55)	ATP-30
Shalaby (46)	Egypt	400	100	178 (44.5 %)	2,4,6,7	21.18 (2.1)	ATP-30
Al Qubtan et al. (51)	Oman	269	90.4	100 (37%)	Mixed	NM	ATP-30
Toudehskchuie et al. (49)	Iran	130	83.07	66 (61.11%)	Pre-clinical	20.36 (2.09)	ATP-30
Toudehskchuie et al. (50)	Iran	220	67	56 (37%)	Mixed	20.84 (2.09)	ATP-30
Khan et al. (44)	Pakistan	281	100	165 (58.3%)	Final year	NM	ATP-30
Syed et al. (27)	Pakistan	635	60	164 (43.0%)	3	21.00 (1.11)	Adapted from Feifel's questionnaire
Maqsood et al. (48)	Pakistan	538	100	240 (44.60%)	1, 4	NM	ATP-30
Al-Ansari et al. (47)	Bahrain	170	82.3	49 (35%)	1, 4, 7	20.57 (2.52)	ATP-30

### Summary of results

A combination of both negative and positive attitudes toward psychiatry was reported in most studies. The overall findings, however, revealed a quite positive attitude. Factors that appeared to positively affect attitude toward psychiatry included involving in a romantic relationship with someone suffering from mental illness, young age, and outside-school exposure to materials and information related to psychiatry. However, the association of attitude toward psychiatry with gender (female), academic year, exposure to psychiatry clerkship, personal history of mental illness and having a relative and/or friend with a psychiatric disease remained controversial between studies. There was not any association between attitude toward psychiatry and having a psychiatrist relative, religiosity, having a relative with alcohol or drug dependency, and significant family problems. Table 2 summarizes the main findings of the reviewed studies, and Table 3 summarizes factors affecting attitudes toward psychiatry. Five studies (43, 45–47, 51) reported both mean scores and standard deviations, using the original version of ATP-30 questionnaire, while the other five used another questionnaire, a modified version of ATP-30 questionnaire or not reported both mean and SD, therefore were excluded from the pool. The pooled result of these five eligible studies indicated that the score of EMR medical students on ATP-30 questionnaire is 104.52 ± 13.75, which is above 90 and indicates a positive attitude. Six studies reported the percentage of students considering psychiatry as their future career (27, 45–47, 49, 50) ranged from 7.6% to 38%, with rates of over 25% in five studies.

**Table 2:** Main findings of the included articles in the review of studies assessing the stigma toward psychiatry among medical students in the Eastern Mediterranean Region.

**First author**	**Mean scale score (SD)**	**Males' mean scale score**	**Females' mean scale score**	**Factors with insignificant association with attitudes**	**Factors with a significant effect on attitudes**	**Limitations**	**Main results**
El Hage et al., (45)	111.95 (12.55)	NM	NM	Gender, religiosity, academic year, studying in private or public universities	Being acquainted with a psychiatric patient, poor psychological well-being	Selection bias leading to a non-representative sample since the questionnaire has been sent electronically to medical students; the lower response rate in sixth and seventh medical students, and low response rate from certain universities, which make the generalization difficult.	Ninety-five percent of the participants had a positive attitude^+^ toward psychiatry, and 26.5% of them considered psychiatry as a potential career choice.
Alzahrani (43)	96.49 (3.30)^*^	94.44 (2.55)	97.77 (3.07)	Having a psychiatrist relative, having a relative with a psychiatric illness	Gender (female), exposure to psychiatric clerkship	Participants from only one institute	There was a general finding of a positive attitude^+^ toward psychiatry. Psychiatry as a future career was still unpopular among male students. Exposure to psychiatry clerkship and gender were the most effective predictors.
Shalaby (46)	99.31 (15.89)	NM	NM	Gender (There was no significant difference between males and females, but females had higher ATP-30 scores)	NA	The study was done in only one Egyptian medical school and at a one time-point. Sampling included only the students in four preselected years and did not include first, third, and fifth-year students	Seventy-six percent of the students had positive attitudes^+^ toward psychiatry and 29.5 % considered psychiatry as a potential career choice.
Al Qubtan et al. (51)	104.20 (12.02)	NM	NM	Gender, experience in the psychiatry rotation, suffering from mental illness, having a relative or friend with mental illness, academic year	NA	Uni-center	The students had an overall positive attitude^+^ toward psychiatry, but none of the studied factors predicted the students' attitude toward psychiatry.
Toudehskchuie et al. (49)	66.00 (8.86)	NM	NM	Gender (No significant difference was reported between males' and females' ATP-30 scores, but females had higher scores)	NA	Uni-center, questionable reliability and validity of the translated questionnaire	Female preclinical students had more positive attitudes than their male counterparts.
Toudehskchuie et al. (50)	Clinical students: 81.55 (12.82)* preclinical students: 62.5 (7.16)	NM	NM	Gender (in pre-clinical students)	NA	Uni-center, questionable reliability and validity of the translated questionnaire	Pre-clinical students generally showed more positive attitudes than their clinical counterparts. In addition, female clinical students had more positive attitudes than their male counterparts.
Khan et al. (44)	100.6^*^	98.56^*^	103.51^*^	Gender	NA	-	The results show an overall positive attitude^+^ of the students toward most aspects of psychiatry.
Syed et al. (27)	NM	NA	NA	NA	NA	-	A small number of students reported psychiatry as their specialty of choice.
Maqsood et al. (48)	98.56^*^	96.7^*^	100.02^*^	NA	NA	Uni-center	The students had a positive attitude^+^ toward psychiatry.
Al-Ansari et al., (47)	105.79 (13.34)	100.63^*^	108.59^*^	Student perception of teaching quality, exposure to clinical psychiatry, visiting the psychiatric hospital, relationship with mental health professionals, having a psychiatric illness, having relative with alcohol or drug dependency, significant family problems, unpleasant experience with mental health personnel	Exposure to material related to psychiatry, having a friend with a psychiatric illness, having a romantic relationship with someone who had a psychiatric illness, young age, gender (female)	-	The students had a moderately positive attitude^+^ toward psychiatry, with a better attitude among female, younger and junior students.

NA, not assessed; NM, not mentioned; ATP, attitudes toward psychiatry; SD: standard deviation.

* Not mentioned explicitly and were calculated by the authors of the present review;

+ according to the ATP-30 questionnaire, scores above 90 indicate a positive attitude.

**Table 3:** Summary of factors affecting attitudes toward psychiatry in the review of studies assessing the stigma toward psychiatry among medical students in the Eastern Mediterranean Region.

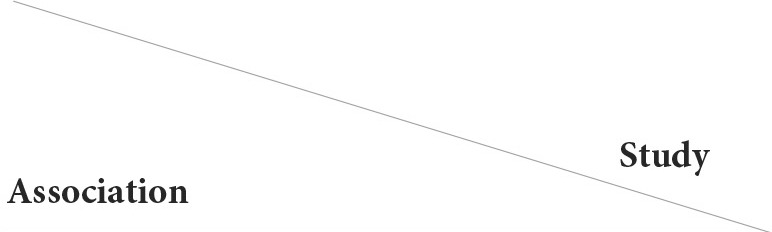	**Hage, 2021**	**Alzahrani, 2019**	**Shalaby, 2016**	**Al Qubtan, 2016**	**Toudehskchuie, 2012**	**Toudehskchuie, 2012**	**Khan, 2008**	**Syed, 2008**	**Maqsood, 2006**	**Al-Ansari, 2002**	**Total Insignificant and Significant**	**Associations (out of 10 studies)**
Gender											6	2
Personal history of mental illness											2	1
Having a relative and/or friend with a mental illness											2	2
Having a relative with drug/alcohol dependency^*^											1	0
Psychiatry clerkship											2	1
Religiosity											1	0
Studying in private or public universities											1	0
Having a psychiatrist relative											1	0
Academic year											2	1
Significant family problems											1	0
Unpleasant experience with mental health											1	0
Romantic relationship with someone having a psychiatric illness											0	1
Unpleasant experience with mental health personnel											1	0
Outside-school exposure to materials related to psychiatry											0	1
Age											0	1
Visiting psychiatric hospital											1	0

Significant association: 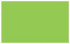 Insignificant association: 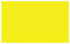 Not studied: 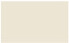.

* Drug/Alcohol dependency can be considered a type of mental illness, but Al-Ansari's study distinguished between drug/alcohol dependency and mental illness.

### Risk of bias assessment

We used JBI Critical Appraisal Checklists for prevalence and analytical cross-sectional studies for bias risk assessment of included studies. Accordingly, one study did not meet the predefined threshold score (the study score = 3) and was excluded due to the risk of bias (52). Supplementary Tables 5, 6 provide a summary of the risk of bias assessments.

## Discussion

### Summary of main findings

With the growing prevalence of psychiatric disorders threatening health systems and an inadequate proportion of medical students choosing psychiatry as a career, special attention should be paid to the students' attitudes toward psychiatry, their career choices, and related trends. This issue is even more crucial for EMR countries, considering the challenges their health systems face. In this regard, we systematically reviewed studies focusing on medical students' attitudes toward psychiatry, associated stigma, and the factors influencing their attitude. This review indicated that the attitudes of EMR medical students toward psychiatry are not stigmatized and are generally positive.

Compared with previous studies documenting negative attitudes toward psychiatry, what has changed over the years might be that various aspects of psychiatry have progressed and the field is rapidly expanding (4, 48, 55): accessible scientific evidence establishing psychotherapeutic treatments' effectiveness; the development of psychosomatic medicine applying to all medical diseases; improved quality of psychiatry teaching; growing prevalence of psychiatric diseases resulting in more exposure to the mentally-ill patients; and involvement with the acutely disturbed as well as successful cases of psychiatry may have exposed students to significance and advantages of psychiatry and challenged commonly held stigmatized view toward it.

In total, studies were originating from 7 different EMR countries (Pakistan, Iran, Saudi Arabia, Lebanon, Egypt, Bahrain, and Oman), lacking data from the other 15 countries. The reviewed studies generally had high response rates, which can be attributed to the participant recruitment method used in most of them, the convenient sampling technique. The results of this review indicate that medical students' attitudes toward psychiatry are generally positive in EMR countries. Our findings regarding attitudes of medical students are consistent with Lyons' (18) review of medical students' attitudes toward psychiatry encompassing 32 studies from 22 different countries across five continents, which demonstrated a generally positive attitude toward psychiatry among medical students of those countries. This is not in line with the traditional view of prevalent stigmatized attitudes in developing countries, especially in Asia (56). Indeed, EMR medical students (ATP-30 score = 104.52 ± 13.75), while generally scoring lower than medical students of developed countries such as Switzerland, Austria, Hungary, and Germany on the ATP-30 questionnaires (European ATP-30 sum score = 111.42 ± 13.35), had comparable attitudes to them (57). However, considering developing countries in other parts of the world, EMR medical students had more positive attitudes toward psychiatry than Ethiopian (ATP-30 score = 52.39 ± 13.2), and Indian students (ATP-30 score = 89.83 ± 11.8) (58, 59). Nevertheless, the same trend was not observed in the developing countries of other parts of the world as Indonesian, South African, Malaysian, and Nigerian medical students had overall the same or comparably more positive attitudes than the EMR countries EMR ATP-30 score = 104.52 ± 13.75 (60–63). This may be partly explained by differences in cultural background and personality traits (64). None of the included studies examined the effect of culture and personality traits on attitudes toward psychiatry. Within EMR, however, higher healthcare financing, Gross Domestic Product (GDP), and even health expenditure as a percentage of GDP did not guarantee better attitudes, as low- or middle-income countries such as Iran, Egypt, Lebanon, and Pakistan generally had the same situation as higher income countries such as Saudi Arabia, Bahrain, and Oman (65, 66).

The impact of having the experience of psychiatry clerkship on students' attitudes was mixed in EMR countries. The reviewed studies did not provide a detailed description of the course and the curriculum, but the quality of the clerkship and the duration of exposure to clerkship might have played a role. The impact of psychiatry clerkship on medical students' attitudes toward psychiatry has been the subject of debate. A review by Lyons indicated a mix of both positive and neutral effects of psychiatry clerkship (18). Among the countries reporting beneficial effects of psychiatry clerkship, no relationship of this outcome with the country's status of culture, general health, or other features was described. In contrast, Farooq et al. (25), in their narrative review of factors attracting medical students toward psychiatry, reported improved attitudes following psychiatry clerkship. However, none of their reviewed studies documented stigma before clerkship. This implies that clerkship further improved previously positive attitudes, rather than reducing stigmatized attitudes. This influential role is supported by Qureshi et al. (67) review of the impact of psychiatry clerkship on attitudes toward psychiatry. Comparably, they indicated improved attitudes after the clerkship, but there was no consistency in the evidence in terms of the long-term effectiveness of the rotation. Students gaining improved attitudes had positively rated the quality of their course, been involved in in-patient care, seen a response to treatment, and received encouragement from consultants during the clerkship.

Gender differences did not predict better attitudes toward psychiatry in our review. In contrast to our results, Velikić (19), in a review of 42 studies from more than 40 countries worldwide, reported female gender as a possible predictor of better attitudes; making a strong prediction, however, may be difficult in the face of much heterogeneity among studies. In consistent with our review, Warnke et al. (57), in their survey of four European countries, have not described a substantial gender-based difference concerning students' attitudes toward psychiatry. This is further supported by the findings of Qureshi et al. (67) review, which reported inconsistency among the studies in the findings of gender correlations.

Not all of the included studies provided the percentage of students who considered psychiatry as their career. Regardless, psychiatry was not an unpopular career choice. This is in contrast to the Nortje et al. (20) review that demonstrated the low popularity of psychiatry as a career among medical students in lower-income countries; however, most of their included studies were not conducted in EMR. In our review, considering psychiatry as a future career was associated with positive attitudes, a finding consistent with that of Nortje et al.

Exposure to mental illness, either self-afflicted or having a friend or relative with mental illness, is another presumed predictor of better attitudes (25). Findings in EMR countries concerning the impact of exposure to mental illness are contradictory. Nevertheless, positive correlations with having a mentally-ill friend or relative could be seen in countries with a relatively higher prevalence of mental illness (29).

## Limitations

Some factors may limit the generalizability of the results of this review. A number of the included studies face limitations regarding their sample sizes and most of them were not multicenter. Furthermore, the small number of available studies and lack of studies from most EMR countries can restrict the results of this review. Therefore, we exercise caution in interpreting the findings of the review and generalizing them. Finally, studies were not homogeneous in terms of their publication year and the results of the review may not reflect the current status of medical students' attitudes in EMR. In addition, none of the included studies addressed effects of socioeconomic factors such as culture and country incomes on attitudes of students toward psychiatry and effects of these factors remain to be explored.

Furthermore, we limited publications to English and Persian languages; however, we did not encounter eligible publications from other languages in our searches. Hence, despite this limitation, the scope of literature in our review remained intact.

## Conclusion

The results of our study showed that the attitudes of EMR medical students toward psychiatry were generally positive. Several factors may mediate their attitudes toward psychiatry, but the study findings were contradictory. These factors included the experience of psychiatry clerkship, history of mental illness or having a relative with mental illness, and female gender. Findings from this review indicated an apparent lack of sufficient information about the attitudes of medical students toward psychiatry in most EMR countries. We suggest regional and cross-country collaborations for greater studies assessing attitudes of medical students toward psychiatry, and special effort is required for the conduction of studies exploring medical students' attitudes toward psychiatry in most of EMR countries lacking such data.

## Protocol deviations

In addition to English publications, we included two Persian studies which is against the registered protocol (Registration 10.17605/OSF.IO/3M2UW), in which non-English language studies planned to be excluded. This happened because these were the only non-English publications we had in our search, and since Persian is the first language of the authors, there was no difficulty in extracting the data.

## Data availability statement

The original contributions presented in the study are included in the article/Supplementary material, further inquiries can be directed to the corresponding author.

## Author contributions

HM and NN designed the study. MM designed the search strategy and ran the search. MM, FG, and NN were involved in the screening, data extraction, and quality assessment process. NN and MM designed the geographical distribution and PRISMA diagram figures respectively. MM and FG designed the tables. HM and MM contributed to the data synthesis. MM, NN, FG, AK, and HM contributed to the protocol development. All authors contributed to the article and approved the submitted version.
