# Supplementary material for: Attitudes of medical students toward psychiatry in Eastern Mediterranean Region: A systematic review
Source: Front Psychiatry. 2023 Jan 26;13:1027377. doi: 10.3389/fpsyt.2022.1027377 (PMC9909281; doi:10.3389/fpsyt.2022.1027377)
Supplement: Supplementary file 1 [file Data_Sheet_1.docx]

Supplementary Material

Supplementary Table 1. Search strategy for PubMed

| #1 | ((("Psychiatry"[MeSH Terms] OR "Psychiatry"[Text Word] OR "mental disorders"[MeSH Terms] OR "mental illness*"[Text Word] OR "psychiatric illness*"[Text Word] OR "psychiatric disorder*"[Text Word] OR "psychiatric disease*"[Text Word]) |
| --- | --- |
| #2 | ("attitude*"[Text Word] OR "stigma*"[Text Word] OR "social stigma"[MeSH Terms] OR "stereotyp*"[Text Word] OR "Prejudice"[Text Word] OR "discrimination"[Text Word])) |
| #3 | ("medical student*"[Text Word] OR "students, medical"[MeSH Terms] OR "extern"[Text Word] OR "externs"[Text Word])) |
| #4 | (Afghanistan OR Bahrain OR Egypt OR Iran OR Iraq OR Jordan OR Kuwait OR Lebanon OR Libya OR Morocco OR Oman OR Pakistan OR Palestine OR "Occupied Palestine Territory" OR Qatar OR "Saudi Arabia" OR Sudan OR "South Sudan" OR Somalia OR “Syrian Arab Republic” OR Syria OR Tunisia OR "United Arab Emirates" OR UAE OR Yemen OR Djibouti) |
| #5 | #1 AND #2 AND #3 AND #4 |

Supplementary Table 2. Search strategy for Scopus

| #1 | TITLE-ABS-KEY (psychiatry OR "mental disorder" OR "mental illness" OR "psychiatric illness" OR "psychiatric disorder" OR "psychiatric disease") |
| --- | --- |
| #2 | TITLE-ABS-KEY (attitude OR stigma* OR stereotyp* OR prejudice OR discrimination) |
| #3 | TITLE-ABS-KEY ("medical student" OR "extern") |
| #4 | ALL (Afghanistan OR Bahrain OR Egypt OR Iran OR Iraq OR Jordan OR Kuwait OR Lebanon OR Libya OR Morocco OR Oman OR Pakistan OR Palestine OR "Occupied Palestine Territory" OR Qatar OR "Saudi Arabia" OR Sudan OR "South Sudan" OR Somalia OR "Syrian Arab Republic" OR Syria OR Tunisia OR "United Arab Emirates" OR UAE OR Yemen OR Djibouti) |
| #5 | #1 AND #2 AND #3 AND #4 |

Supplementary Table 3. Search strategy for Web of Science

| #1 | TS= (psychiatry OR "mental disorder*" OR "mental illness*" OR "psychiatric illness*" OR "psychiatric disorder*" OR "psychiatric disease*") |
| --- | --- |
| #2 | TS= (attitude* OR stigma* OR Stereotyp* OR Prejudice OR discrimination) |
| #3 | TS= ("medical student*" OR "extern$") |
| #4 | ALL= (Afghanistan OR Bahrain OR Egypt OR Iran OR Iraq OR Jordan OR Kuwait OR Lebanon OR Libya OR Morocco OR Oman OR Pakistan OR Palestine OR “Occupied Palestine Territory” OR Qatar OR "Saudi Arabia" OR Sudan OR "South Sudan" OR Somalia OR “Syrian Arab Republic” OR Syria OR Tunisia OR "United Arab Emirates" OR UAE OR Yemen OR Djibouti) |
| #5 | #1 AND #2 AND #3 AND #4 |

Supplementary Table 4. Search strategy for PsychInfo (PsychARTICLES)

| #1 | Any Field: psychiatry OR Any Field: "mental disorder*" OR Any Field: "mental illness*" OR Any Field: "psychiatric illness*" OR Any Field: "psychiatric disorder*" OR Any Field: "psychiatric disease*" |
| --- | --- |
| #2 | Any Field: attitude* OR Any Field: stigma* OR Any Field: Stereotyp* OR Any Field: Prejudice OR Any Field: discrimination |
| #3 | Any Field: "medical student*" OR Any Field: "extern*" |
| #4 | Any Field: Afghanistan OR Any Field: Egypt OR Any Field: Iran OR Any Field: Iraq OR Any Field: Jordan OR Any Field: Kuwait OR Any Field: Lebanon OR Any Field: Libya OR Any Field: Morocco OR Any Field: Oman OR Any Field: Pakistan OR Any Field: Palestine OR Any Field: Qatar OR Any Field: "Saudi Arabia" OR Any Field: Sudan OR Any Field: "South Sudan" OR Any Field: Somalia OR Any Field: Syria OR Any Field: “Syrian Arab Republic” OR Any Field: Tunisia OR Any Field: "United Arab Emirates" OR Any Field: Bahrain OR Any Field: UAE OR Any Field: Yemen OR Any Field: Djibouti |
| #5 | #1 AND #2 AND #3 AND #4 |

Supplementary Table 5. Quality assessment of prevalence studies using Joanna Briggs Institute checklist for prevalence studies

| **Included studies** | **Q1** | **Q2** | **Q3** | **Q4** | **Q5** | **Q6** | **Q7** | **Q8** | **Q9** | **Total** |
| --- | --- | --- | --- | --- | --- | --- | --- | --- | --- | --- |
| A S Shalaby, 2016 | Y | Y | Y | Y | Y | Y | U | Y | Y | 8 |
| G G Toudehskchuie, 2012 | Y | Y | Y | Y | U | Y | U | Y | Y | 7 |
| G G Toudehskchuie, 2012 | Y | Y | Y | Y | U | Y | U | Y | Y | 7 |
| S A Khan, 2008 | Y | Y | Y | Y | U | Y | U | U | Y | 6 |
| E U Syed, 2008 | Y | Y | Y | Y | U | Y | U | Y | Y | 7 |
| N Maqsood, 2006 | Y | Y | Y | Y | U | Y | U | U | Y | 6 |

N, NO; Y, yes; U, Unclear

C*riteria for the critical appraisal qualitative evidence*

*Q1 =* *Was the sample frame appropriate to address the target population?*

*Q2 = Were study participants sampled in an appropriate way?*

*Q3 = Was the sample size adequate?*

*Q4 = Were the study subjects and the setting described in detail?*

*Q5 = Was the data analysis conducted with sufficient coverage of the identified sample?*

*Q6 = Were valid methods used for the identification of the condition?*

*Q7 = Was the condition measured in a standard, reliable way for all participants?*

*Q8 = Was there appropriate statistical analysis?*

*Q9 = Was the response rate adequate, and if not, was the low response rate managed appropriately?*

Supplementary Table 6. Quality assessments of analytical cross-sectional studies using the Joanna Briggs Institute checklist for analytical cross-sectional studies

| **Total** | **Q8** | **Q7** | **Q6** | **Q5** | **Q4** | **Q3** | **Q2** | **Q1** | Included studies |
| --- | --- | --- | --- | --- | --- | --- | --- | --- | --- |
| 5 | Y | Y | NA | N | NA | Y | Y | Y | S E Hage, 2021 |
| 4 | Y | Y | NA | N | NA | Y | Y | N | A Alzahrani, 2019 |
| 5 | Y | Y | NA | N | NA | Y | Y | Y | A Al Qubtan, 2016 |
| 3 | Y | Y | NA | N | NA | NA | Y | N | K Farooq, 2014 |
| 5 | Y | Y | NA | N | NA | Y | Y | Y | A Al-Ansari, 2002 |

*N, NO; Y, yes; NA, No assessment.*

*Criteria for the critical appraisal of qualitative evidence:*

*Q1 = Were the criteria for inclusion in the sample clearly defined?*

*Q2 = Were the study subjects and the setting described in detail?*

*Q3 = Was the exposure measured in a valid and reliable way?*

*Q4 = Were objective, standard criteria used for measurement of the condition?*

*Q5 = Were confounding factors identified?*

*Q6 = Were strategies to deal with confounding factors stated?*

*Q7 = Were the outcomes measured in a valid and reliable way?*

*Q8 = Was appropriate statistical analysis used?*
